# Benefits of budesonide/glycopyrronium/formoterol fumarate dihydrate on lung function and exacerbations of COPD: a post-hoc analysis of the KRONOS study by blood eosinophil level and exacerbation history

**DOI:** 10.1186/s12931-024-02918-8

**Published:** 2024-08-05

**Authors:** Shigeo Muro, Tomotaka Kawayama, Hisatoshi Sugiura, Munehiro Seki, Elizabeth A. Duncan, Karin Bowen, Jonathan Marshall, Ayman Megally, Mehul Patel

**Affiliations:** 1 Department of Respiratory Medicine, Nara Medical University, 840 Shijo-cho, Nara, 634-8522 Japan; 2 Division of Respirology, Neurology and Rheumatology, Department of Medicine, Kurume University School of Medicine, Kurume, Japan; 3 Department of Respiratory Medicine, Tohoku University Graduate School of Medicine, Sendai, Japan; 4 Respiratory Inhalation, Medical Department, AstraZeneca K.K. Kita-ku, Osaka, Japan; 5 Formerly of AstraZeneca, Durham, NC USA; 6 Late RIA Biometrics, AstraZeneca, Gaithersburg, MD USA; 7 BioPharmaceuticals, R&I Medical, AstraZeneca, Cambridge, UK; 8 Late RIA, BioPharmaceuticals R&D, AstraZeneca, Gaithersburg, MD USA; 9 Late RIA, BioPharmaceuticals R&D, AstraZeneca, Cambridge, UK

**Keywords:** Blood eosinophils, Budesonide/glycopyrronium/formoterol fumarate dihydrate (BGF), Chronic obstructive pulmonary disease (COPD), Disease severity, Exacerbation rates, Lung function, Fixed-dose triple therapy

## Abstract

**Background:**

Japanese guidelines recommend triple inhaled corticosteroid (ICS)/long-acting muscarinic antagonist (LAMA)/long-acting β_2_-agonist (LABA) therapy in patients with chronic obstructive pulmonary disease (COPD) and no concurrent asthma diagnosis who experience frequent exacerbations and have blood eosinophil (EOS) count ≥ 300 cells/mm^3^, and in patients with COPD and asthma with continuing/worsening symptoms despite receiving dual ICS/LABA therapy. These post-hoc analyses of the KRONOS study in patients with COPD and without an asthma diagnosis, examine the effects of fixed-dose triple therapy with budesonide/glycopyrronium/formoterol fumarate dihydrate (BGF) versus dual therapies on lung function and exacerbations based on blood EOS count – focusing on blood EOS count 100 to < 300 cells/mm^3^ – as a function of exacerbation history and COPD severity.

**Methods:**

In KRONOS, patients were randomized to receive treatments that included BGF 320/14.4/10 µg, glycopyrronium/formoterol fumarate dihydrate (GFF) 14.4/10 µg, or budesonide/formoterol fumarate dihydrate (BFF) 320/10 µg via metered dose inhaler (two inhalations twice-daily for 24 weeks). These post-hoc analyses assessed changes from baseline in morning pre-dose trough forced expiratory volume in 1 s (FEV_1_) over 12–24 weeks and moderate or severe COPD exacerbations rates over 24 weeks. The KRONOS study was not prospectively powered for these subgroup analyses.

**Results:**

Among patients with blood EOS count 100 to < 300 cells/mm^3^, least squares mean treatment differences for lung function improvement favored BGF over BFF in patients without an exacerbation history in the past year and in patients with moderate and severe COPD, with observed differences ranging from 62 ml to 73 ml across populations. In this same blood EOS population, moderate or severe exacerbation rates were reduced for BGF relative to GFF by 56% in patients without an exacerbation history in the past year, by 47% in patients with moderate COPD, and by 50% in patients with severe COPD.

**Conclusions:**

These post-hoc analyses of patients with moderate-to-very severe COPD from the KRONOS study seem to indicate clinicians may want to consider a step-up to triple therapy in patients with persistent/worsening symptoms with blood EOS count > 100 cells/mm^3^, even if disease severity is moderate and there is no recent history of exacerbations.

**Trial registration:**

ClinicalTrials.gov registry number NCT02497001 (registration date, 13 July 2015).

**Supplementary Information:**

The online version contains supplementary material available at 10.1186/s12931-024-02918-8.

## Background

Chronic obstructive pulmonary disease (COPD) is a leading cause of morbidity and mortality worldwide, with economic and social burdens that are both substantial and increasing [1, 2]. Three fixed-dose triple therapies with an inhaled corticosteroid (ICS), a long-acting muscarinic antagonist (LAMA), and a long-acting β_2_-agonist (LABA) are approved for the maintenance treatment of COPD [3–5].

The Global Initiative For Chronic Obstructive Lung Disease (GOLD) 2023 report recommends triple therapy with an ICS/LAMA/LABA be considered as an initial treatment option in patients with blood eosinophil (EOS) count ≥ 300 cells/mm^3^ with frequent (≥ 2/year) moderate exacerbations or (≥ 1) exacerbation leading to hospitalization [2]. A step up to ICS/LAMA/LABA triple therapy is also recommended in patients with blood EOS count ≥ 100 cells/mm^3^ who experience exacerbations despite receiving LAMA/LABA dual therapy [2]. According to COPD treatment guidelines in Japan [6], ICS-containing treatment is recommended for patients with COPD and a clinical asthma diagnosis when dual therapy is not sufficient; however, in patients with COPD and no asthma diagnosis, ICS/LAMA/LABA triple therapy is only recommended for those who experience frequent exacerbations (≥ 2 moderate or ≥ 1 severe per year) and have blood EOS count ≥ 300 cells/mm^3^.

In ETHOS (NCT02465567), a study of patients with moderate-to-very severe COPD with exacerbations and receiving at least two inhaled maintenance therapies at screening, the fixed-dose triple combination therapy budesonide/glycopyrronium/formoterol fumarate dihydrate (BGF) 320/14.4/10 µg significantly reduced the annual rate of moderate or severe exacerbations (the primary study end point) [7] and significantly improved lung function (pulmonary function test sub-study primary endpoint) versus glycopyrronium/formoterol fumarate dihydrate (GFF) and budesonide/formoterol fumarate dihydrate (BFF) [8]. Similarly, in KRONOS (NCT02497001), a study of patients with moderate-to-very severe COPD and no requirement for prior exacerbations, BGF 320/14.4/10 significantly improved lung function versus GFF, BFF, and open-label budesonide/formoterol fumarate dry-powder inhaler (BUD/FORM), and significantly reduced the rate of moderate or severe exacerbations versus GFF [9].

Importantly, benefits of BGF over dual therapy were observed across a range of blood EOS counts in post-hoc analyses of ETHOS and KRONOS [10–12]. Given recommendations in the GOLD 2023 report [2], current Japanese treatment guidelines [6], and evidence for benefits of BGF over dual therapy across a range of blood EOS counts (including below 300 cells/mm^3^) in patients with COPD [10, 11], post-hoc analyses of the KRONOS study were conducted to further examine the effects of BGF versus dual LAMA/LABA and ICS/LABA therapies on lung function and exacerbation rates in patients with COPD based on blood EOS count (100 to < 300 and ≥ 100 cells/mm³) as a function of exacerbation history (exacerbations in the past year and no exacerbations in the past year) and COPD severity (moderate, severe, very severe).

## Methods

### Study design

A detailed description of the study design and patient population in KRONOS (ClinicalTrials.gov registry number NCT02497001; registration date, 13 July 2015), including inclusion and exclusion criteria, has been previously published [9]. In brief, KRONOS was a 24-week, double-blind, parallel-group, phase III randomized controlled study conducted at 215 sites across four countries (Canada, China, Japan, and the United States).

At screening, eligible patients discontinued current COPD medications (i.e., LAMA, LABA, or both) for the study duration and received open-label ipratropium bromide four times daily as COPD maintenance therapy. ICS use was permitted during screening, provided patients were on a stable dose for at least 4 weeks before screening; however, both ipratropium and ICS were stopped before randomization. Rescue use of salbutamol was permitted throughout the study.

After screening, patients were randomized 2:2:1:1 to receive BGF 320/14.4/10 µg, GFF 14.4/10 µg, or BFF 320/10 µg via a single Aerosphere^™^ metered dose inhaler, or open-label BUD/FORM 400/12 µg via a dry powder inhaler (Symbicort^®^ Turbuhaler^®^), as two inhalations twice-daily for 24 weeks. As BFF was not an approved COPD therapy at the time KRONOS was conducted, BUD/FORM (which was already approved for COPD treatment) was included as an active comparator to support BFF as a comparator for BGF. However, for the purposes of this post-hoc analysis, only data for BFF and GFF are reported.

The study was conducted in accordance with Good Clinical Practice, including the Declaration of Helsinki. The protocol and informed consent form were approved by appropriate institutional review boards or independent ethics committees prior to the start of the study (a full listing of appropriate institutional review boards or independent ethics committees has been published [9]). All patients provided written informed consent before screening.

### Patients

Key inclusion criteria for the KRONOS study have been described in detail previously [9]. Eligible patients were aged 40–80 years; were current or former smokers (smoking history of ≥ 10 pack-years); had an established COPD clinical history, as defined by the American Thoracic Society/European Respiratory Society [13] or Japanese local guidelines [14]; had moderate-to-very severe COPD, defined as post-bronchodilator FEV_1_ of 25–80% of predicted normal values based on National Health and Nutrition Examination Survey III reference equations [15] or applicable local reference norms [14–17]; and were symptomatic (as defined by a COPD Assessment Test score ≥ 10) despite treatment with ≥ 2 inhaled maintenance therapies for ≥ 6 weeks before screening. Patients were not required to have a history of COPD exacerbations in the previous 12 months and were excluded if they had a current diagnosis of asthma or any respiratory disease other than COPD, evaluated by the investigator, that could affect study results.

### Outcomes

In the KRONOS study, the primary lung function endpoint, according to the Japanese/Chinese regulatory approach, was change from baseline in morning pre-dose trough FEV_1_ over 12–24 weeks; the rate of moderate or severe COPD exacerbations over 24 weeks was a secondary efficacy endpoint [12].

A COPD exacerbation was defined as a change in the patient’s usual COPD symptoms lasting for ≥ 2 days that was beyond normal day-to-day variation, acute in onset, and may have warranted a change in regular medication. An exacerbation was considered moderate if it resulted in systemic corticosteroid and/or antibiotic use for at least 3 days, and as severe if it resulted in an inpatient COPD-related hospitalization or death.

### Data presentation and statistical analyses

For the current post-hoc analyses, change from baseline in morning pre-dose trough FEV_1_ over 12–24 weeks and the rate of moderate or severe COPD exacerbations over 24 weeks were analyzed in patients with blood EOS counts of 100 to < 300 cells/mm^3^ and ≥ 100 cells/mm^3^ as a function of exacerbation history (any moderate or severe exacerbations in the past year; no exacerbations in the past year) and COPD severity (moderate [FEV_1_ 50–<80% predicted], severe [FEV_1_ 30–<50% predicted], very severe [FEV_1_ < 30% predicted]). Analyses were conducted in the modified intention-to-treat (mITT) population, which included all patients with post-randomization data obtained before treatment discontinuation.

The primary baseline EOS subgroup of interest included those with blood EOS count 100 to < 300 cells/mm^3^, as assessment of this subgroup will provide insight into the benefits of BGF among patients with blood EOS count < 300 cells/mm^3^. The blood EOS count ≥ 100 cells/mm^3^ subgroup was included to provide supportive evidence that inclusion of patients with blood EOS count > 300 cells/mm^3^ in the analysis did not result in substantively different findings. Patients with blood EOS count < 100 cells/mm^3^ were not included in the post-hoc analyses because the population size would be small and the published literature supports greater ICS benefits with higher EOS count [7, 9, 18–21] and lesser ICS efficacy with low blood EOS count [2, 8, 20].

Demographic and clinical characteristics are reported descriptively across treatment arms for each subgroup. Change from baseline in morning pre-dose trough FEV_1_ over 12–24 weeks in each EOS subgroup by exacerbation history in the preceding 12 months or COPD severity was assessed using a linear repeated measures model that included baseline FEV_1_, percent reversibility to salbutamol, and baseline blood EOS count as continuous covariates and visit, treatment, treatment-by-visit interaction, and ICS use at screening (yes or no), as categorical covariates. Data reported includes the least squares (LS) mean change from baseline with 95% confidence intervals (CIs) for each treatment and LS mean differences with 95% CIs in the change from baseline for each treatment versus BGF.

The rate of moderate or severe exacerbations over 24 weeks in each EOS subgroup by exacerbation history in the preceding 12 months or COPD severity was assessed using negative binomial regression; treatments were compared with adjustment for baseline post-bronchodilator percent predicted FEV_1_, baseline COPD exacerbation history (0, 1, or ≥ 2) in the preceding 12 months, log baseline blood EOS count, region, and ICS use at screening (yes or no). The logarithm of the time at risk of experiencing an exacerbation was used as an offset variable in the model. The data reported includes the number (%) of patients with exacerbations, the total time at risk for an exacerbation, and the adjusted (standard error [SE]) rate of moderate or severe exacerbations; treatment differences between BGF and the other treatment arms are reported using rate ratios (RR) with 95% CIs. As the KRONOS study was not prospectively powered for any of the reported post-hoc analyses, reported *P*-values are nominal, unadjusted for multiplicity, and provided for descriptive purposes only.

## Results

### Patient disposition and characteristics

The disposition and demographic/clinical characteristics of patients in the KRONOS study has been described in detail previously [9]. In brief, of 1902 randomized patients, 1896 were included in the mITT population (BGF, *n* = 639; GFF, *n* = 625; BFF, *n* = 314). Across treatment groups in the overall mITT population, the average age was approximately 65 years, and the median blood EOS count was approximately 150 cells/mm^3^; approximately 74% of patients did not report having an exacerbation in the preceding 12 months.

Demographic and clinical characteristics in patients with blood EOS count 100 to < 300 cells/mm^3^ with and without exacerbations in the preceding 12 months are summarized in Table 1 and in patients categorized based on COPD severity in Additional file 1 supplementary Table S1. Across treatment groups, demographic and clinical characteristics within each exacerbation history subgroup and COPD severity subgroup were well balanced, with the exception of those variables associated with categorization (i.e., exacerbation history or FEV_1_% predicted). Similarly, among patients with blood EOS count ≥ 100 cells/mm^3^, patient characteristics in each exacerbation history subgroup (Additional file 1 supplementary Table S2) or COPD severity subgroup (Additional file 1 supplementary Table S3) were also well balanced across treatment groups.

**Table 1:** Demographic and clinical characteristics: EOS count 100 to < 300 cells/mm^3^ by exacerbation history, mITT population

	BGF 320/14.4/10 µg	GFF 14.4/10 µg	BFF 320/10 µg
***EOS 100 to < 300 cells/mm*** ^***3***^ *** – without exacerbation history in the preceding 12 months***	***N*** ** = 308**	***N*** ** = 307**	***N*** ** = 147**
** Mean (SD) age**,** years**^**a**^	65.2 (7.9)	64.9 (7.8)	66.0 (7.1)
** Sex**,** male n (%)**	207 (67.2)	204 (66.4)	110 (74.8)
** EOS count**			
Median (range) cells per mm^3^	170.0 (100.0–295.0)	165.0 (100.0–295.0)	175.0 (100.0–295.0)
≥ 150 cells/mm^3^, n (%)	190 (61.7)	188 (61.2)	100 (68.0)
** Current smoker**,** n (%)**	136 (44.2)	129 (42.0)	56 (38.1)
** Mean (SD) number of pack-years smoked** ^**b**^	56.6 (32.4)	51.2 (27.0)	51.4 (26.4)
** Mean (SD) post-salbutamol FEV**_**1**_, **% predicted**	51.1 (13.8)	51.6 (13.6)	50.5 (13.5)
** Moderate or severe COPD exacerbations in the past 12 months**,** n (%)**			
0	308 (100)	307 (100)	147 (100)
1	0	0	0
≥ 2	0	0	0
** Used ICS at screening**,** n (%)**	207 (67.2)	206 (67.1)	102 (69.4)
** COPD severity**,** n (%)**			
Mild	0	0	1 (0.7)
Moderate	157 (51.0)	166 (54.1)	70 (47.6)
Severe	131 (42.5)	121 (39.4)	70 (47.6)
Very severe	20 (6.5)	20 (6.5)	6 (4.1)
** Mean (SD) total CAT score** ^**c**^	18.7 (6.6)	18.3 (6.2)	18.6 (6.8)
** Reversibility**^**d**^, **n (%)**	137 (44.5)	136 (44.3)	66 (44.9)
**EOS 100 to < 300 cells/mm** ^**3**^ **– with exacerbation history in the preceding 12 months**	***N*** ** = 102**	***N*** ** = 92**	***N*** ** = 45**
** Mean (SD) age**,** years**^**a**^	62.9 (8.7)	64.8 (8.0)	62.8 (7.2)
** Sex**,** n (%) male**	67 (65.7)	56 (60.9)	26 (57.8)
** EOS count**			
Median (range) cells per mm^3^	165.8 (100.0–295.0)	165.0 (105.0–295.0)	150.0 (100.0–290.0)
≥ 150 cells/mm^3^, n (%)	63 (61.8)	61 (66.3)	23 (51.1)
** Current smoker**,** n (%)**	42 (41.2)	39 (42.4)	21 (46.7)
** Mean (SD) number of pack-years smoked** ^**b**^	48.5 (25.7)	49.8 (25.8)	56.0 (36.0)
** Mean (SD) post-salbutamol FEV**_**1**_, **% predicted**	49.3 (13.2)	47.7 (14.3)	49.1 (16.1)
** Moderate or severe COPD exacerbations in the past 12 months**,** n (%)**			
0	0	0	0
1	77 (75.5)	67 (72.8)	34 (75.6)
≥ 2	25 (24.5)	25 (27.2)	11 (24.4)
** Used ICS at screening**,** n (%)**	76 (74.5)	67 (72.8)	36 (80.0)
** COPD severity**,** n (%)**			
Mild	1 (1.0)	0	0
Moderate	44 (43.1)	38 (41.3)	20 (44.4)
Severe	51 (50.0)	42 (45.7)	19 (42.2)
Very severe	6 (5.9)	12 (13.0)	6 (13.3)
** Mean (SD) total CAT score** ^**c**^	19.5 (6.3)	18.5 (6.5)	19.3 (6.2)
** Reversibility**^**d**^, **n (%)**	50 (49.0)	26 (28.3)	20 (44.4)

^a^Age is the age at the time of informed consent

^b^(Number of cigarettes per day / 20) × number of years smoked

^c^The total score is the sum of eight CAT item scores

^d^Reversibility defined as improvement in FEV_1_ after salbutamol administration (compared with before salbutamol administration) of 12% or more and 200 mL or more

Abbreviations. BFF: budesonide/formoterol fumarate dihydrate; BGF: budesonide/glycopyrronium/formoterol fumarate dihydrate; CAT: COPD Assessment Test; COPD: chronic obstructive pulmonary disease; EOS: eosinophil; FEV_1_: forced expiratory volume in 1 s; GFF: glycopyrronium/formoterol fumarate dihydrate; ICS: inhaled corticosteroid; mITT: modified intention-to-treat; SD: standard deviation

### Lung function

**Fig. 1:**
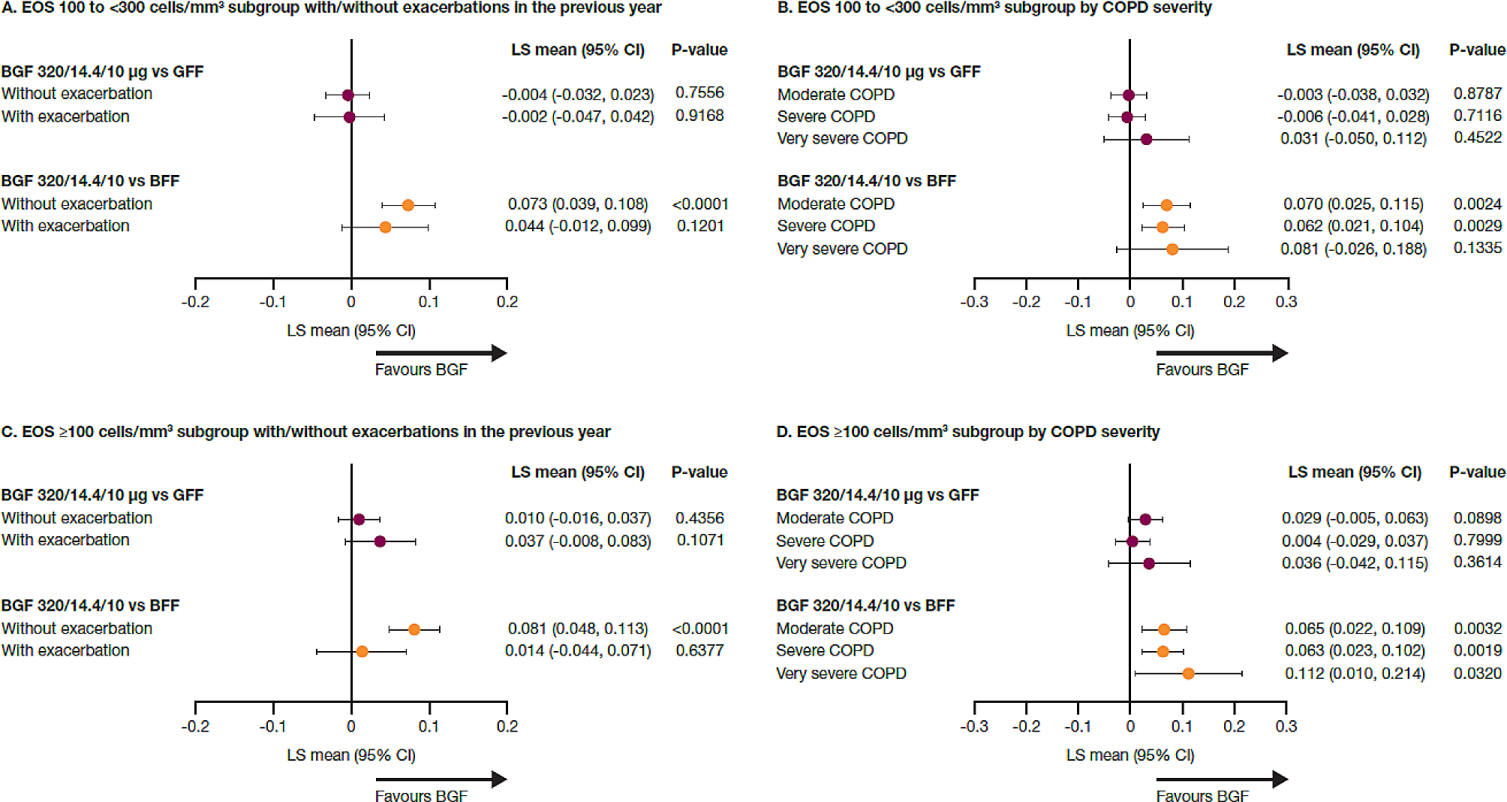
Lung function difference versus BGF^a, b^: EOS subgroups by exacerbation history or COPD severity, mITT population. ^a^Change from baseline in morning pre-dose trough FEV_1_ over 12–24 weeks. ^b^From a linear repeated measures model which included the following covariates: baseline FEV_1_, percent reversibility to salbutamol, and baseline EOS count as continuous covariates and visit, treatment, treatment-by-visit interaction, and ICS use at screening (yes/no) as categorical covariates. Abbreviations: BFF: budesonide/formoterol fumarate dihydrate; BGF: budesonide/glycopyrronium/formoterol fumarate dihydrate; CI: confidence interval; COPD: chronic obstructive pulmonary disease; EOS: eosinophil; FEV_1_: forced expiratory volume in 1 s; GFF: glycopyrronium/formoterol fumarate dihydrate; ICS: inhaled corticosteroid; LS: least squares; mITT: modified intention-to-treat

Across treatment groups, increases from baseline in morning pre-dose trough FEV_1_ were observed over 12–24 weeks for all blood EOS counts by exacerbation history and COPD severity subgroups (Additional file 1 supplementary Table S4). Among patients with blood EOS count 100 to < 300 cells/mm^3^, improvement in lung function with BGF versus BFF was observed among those without an exacerbation history in the preceding 12 months (nominal *P* < 0.0001; Fig. 1A); treatment differences in the changes from baseline in morning pre-dose trough FEV_1_ were not suggestive of differences between BGF and GFF (Fig. 1A). Improvements in lung function with BGF versus BFF were observed among those with moderate and severe COPD (both nominal *P* < 0.05; Fig. 1B), with a similar trend among those with very severe COPD; treatment differences in the changes from baseline in morning pre-dose trough FEV_1_ were not suggestive of differences between BGF and GFF (Fig. 1B).

Similarly, among patients with blood EOS count ≥ 100 cells/mm^3^, improvements in lung function with BGF versus BFF were observed among those without an exacerbation history in the preceding 12 months (nominal *P* < 0.0001; Fig. 1C); treatment differences in the change from baseline in morning pre-dose trough FEV_1_ were not suggestive of differences between BGF and GFF (Fig. 1C). Improvement in lung function with BGF versus BFF was observed regardless of COPD severity (all nominal *P* < 0.05; Fig. 1D). Treatment differences in the change from baseline in morning pre-dose trough FEV_1_ were not suggestive of differences between BGF and GFF in any COPD severity subgroup (Fig. 1D).

### Exacerbation rates

**Fig. 2:**
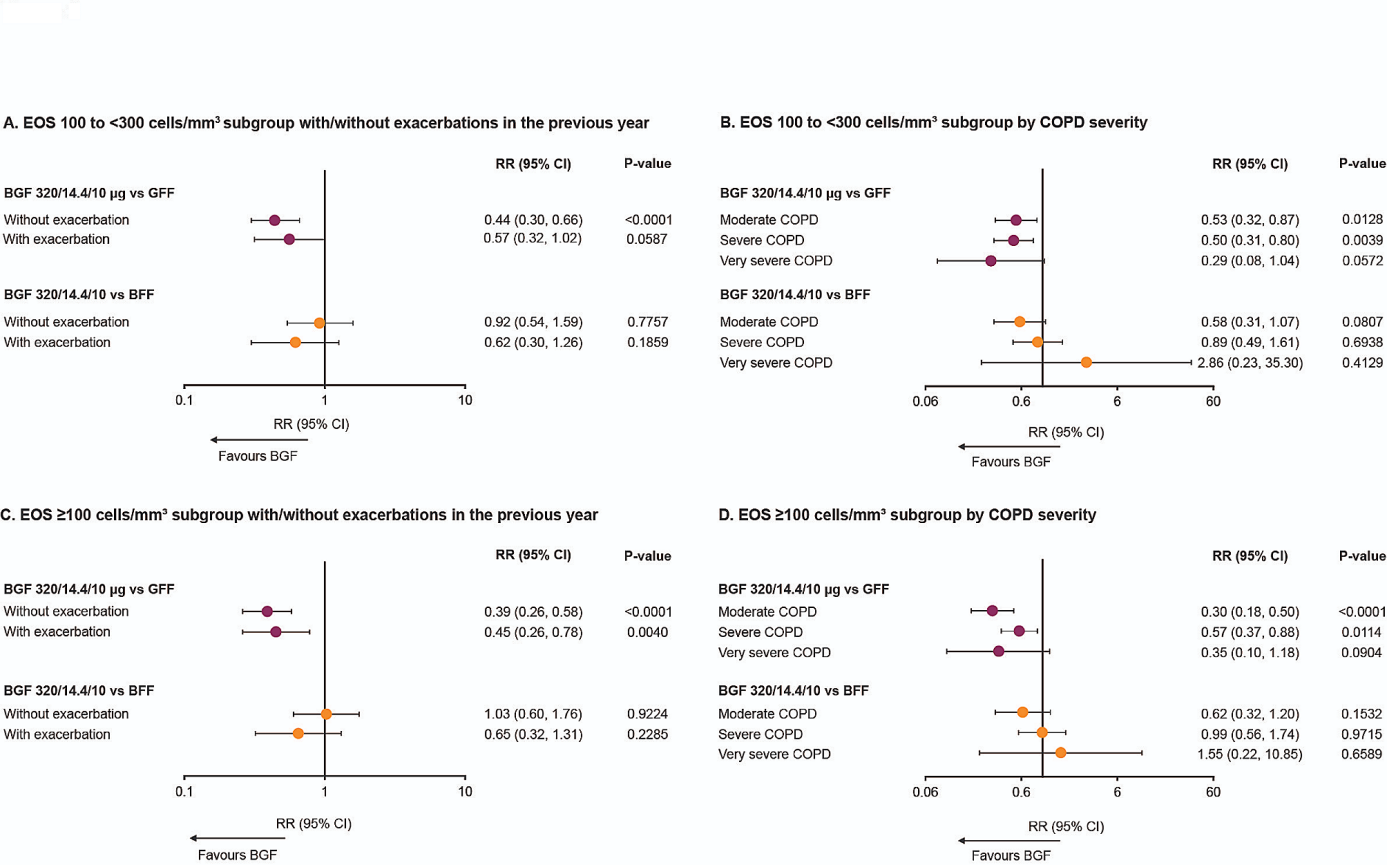
Moderate/severe exacerbation risk versus BGF^a^: EOS subgroups by exacerbation history or COPD severity, mITT population. ^a^ Treatments compared adjusting for baseline post-bronchodilator percent predicted FEV_1_, baseline COPD exacerbation history (0, 1, or ≥ 2) in the preceding 12 months, log baseline blood EOS count, region, and ICS use at screening (yes/no) using negative binomial regression; the logarithm of the time at risk of experiencing an exacerbation was used as an offset variable in the model. Abbreviations: BFF: budesonide/formoterol fumarate dihydrate; BGF: budesonide/glycopyrronium/formoterol fumarate dihydrate; CI: confidence interval; COPD: chronic obstructive pulmonary disease; EOS: eosinophil; FEV_1_: forced expiratory volume in 1 s; GFF: glycopyrronium/formoterol fumarate dihydrate; ICS: inhaled corticosteroid; mITT: modified intention-to-treat; RR: rate ratio

Across blood EOS counts by exacerbation history in the preceding 12 months or COPD severity, the adjusted rate of moderate or severe exacerbations was greater with GFF than any other treatment (Table 2). Among patients with blood EOS count 100 to < 300 cells/mm^3^, the risk of moderate or severe exacerbations was 56% lower for BGF versus GFF in patients without exacerbation history in the preceding 12 months (nominal *P* < 0.0001; Fig. 2A), with a similar trend observed in those with exacerbation history in the preceding 12 months. Risk of moderate or severe exacerbations were 47% and 50% lower, respectively, for BGF versus GFF in patients with moderate and severe COPD (both nominal *P* < 0.05; Fig. 2B), with a similar trend observed for very severe COPD. Examination of RRs for moderate or severe exacerbations between BGF versus BFF was not suggestive of treatment differences for either exacerbation history subgroup (Fig. 2A) or COPD severity group (Fig. 2B).

**Table 2:** Moderate or severe exacerbations: EOS count subgroups by exacerbation history and COPD severity, mITT population

	BGF 320/14.4/10 µg	GFF 14.4/10 µg	BFF 320/10 µg
***EOS 100 to < 300 cells/mm*** ^***3***^			
***Without exacerbation history in the preceding 12 months***	***N*** ** = 308**	***N*** ** = 307**	***N*** ** = 147**
﻿Patients with exacerbations, n (%)	45 (14.6)	76 (24.8)	24 (16.3)
Events^a^	50	99	25
Total time at risk, years	131.31	126.63	61.30
Adjusted COPD exacerbation rate (SE)^b^	0.35 (0.06)	0.79 (0.10)	0.38 (0.09)
***With exacerbation history in the preceding 12 months***	***N*** ** = 102**	***N*** ** = 92**	***N*** ** = 45**
Patients with exacerbations, n (%)	22 (12.6)	32 (34.8)	16 (35.6)
Events^a^	31	41	19
Total time at risk, years	43.51	37.14	17.61
Adjusted COPD exacerbation rate (SE)^b^	0.64 (0.14)	1.13 (0.23)	1.04 (0.31)
***Moderate COPD***	***N*** ** = 201**	***N*** ** = 204**	***N*** ** = 90**
Patients with exacerbations, n (%)	25 (12.4)	44 (21.6)	17 (18.9)
Events^a^	29	50	20
Total time at risk, years	86.54	86.71	36.38
Adjusted COPD exacerbation rate (SE)^b^	0.29 (0.06)	0.55 (0.09)	0.51 (0.13)
***Severe COPD***	***N*** ** = 182**	***N*** ** = 163**	***N*** ** = 89**
Patients with exacerbations, n (%)	37 (20.3)	52 (31.9)	22 (24.7)
Events^a^	47	66	23
Total time at risk, years	77.54	65.04	37.12
Adjusted COPD exacerbation rate (SE)^b^	0.55 (0.10)	1.10 (0.18)	0.62 (0.16)
***Very severe COPD***	***N*** ** = 26**	***N*** ** = 32**	***N*** ** = 12**
Patients with exacerbations, n (%)	5 (19.2)	12 (37.5)	1 (8.3)
Events^a^	5	24	1
Total time at risk, years	10.27	12.01	4.94
Adjusted COPD exacerbation rate (SE)^b^	0.43 (0.24)	1.47 (0.50)	0.15 (0.17)
***EOS ≥ 100 cells/mm*** ^***3***^			
***Without exacerbation history in the preceding 12 months***	***N*** ** = 363**	***N*** ** = 368**	***N*** ** = 179**
Patients with exacerbations, n (%)	51 (14.0)	90 (24.5)	27 (15.1)
Events^a^	60	128	28
Total time at risk, years	155.06	149.63	74.46
Adjusted COPD exacerbation rate (SE)^b^	0.36 (0.057)	0.93 (0.117)	0.35 (0.081)
***With exacerbation history in the preceding 12 months***	***N*** ** = 119**	***N*** ** = 116**	***N*** ** = 53**
Patients with exacerbations, n (%)	27 (22.7)	43 (37.1)	18 (34.0)
Events^a^	37	64	22
Total time at risk, years	50.55	46.12	21.25
Adjusted COPD exacerbation rate (SE)^b^	0.69 (0.148)	1.53 (0.278)	1.07 (0.310)
***Moderate COPD***	***N*** ** = 245**	***N*** ** = 250**	***N*** ** = 113**
Patients with exacerbations, n (%)	29 (11.8)	62 (24.8)	19 (16.8)
Events^a^	34	90	23
Total time at risk, years	105.78	103.10	46.29
Adjusted COPD exacerbation rate (SE)^b^	0.28 (0.06)	0.92 (0.14)	0.45 (0.12)
***Severe COPD***	***N*** ** = 205**	***N*** ** = 198**	***N*** ** = 104**
Patients with exacerbations, n (%)	43 (21.0)	59 (29.8)	24 (23.1)
Events^a^	57	78	25
Total time at risk, years	87.15	78.77	43.16
Adjusted COPD exacerbation rate (SE)^b^	0.60 (0.10)	1.05 (0.16)	0.61 (0.15)
***Very severe COPD***	***N*** ** = 30**	***N*** ** = 36**	***N*** ** = 14**
Patients with exacerbations, n (%)	6 (20.0)	12 (33.3)	2 (14.3)
Events^a^	6	24	2
Total time at risk, years	11.75	13.87	5.80
Adjusted COPD exacerbation rate (SE)^b^	0.45 (0.25)	1.30 (0.46)	0.29 (0.24)

^a^COPD exacerbations were considered separate events provided that 7 or more days were between the recorded stop date of the earlier event and start date of the later

^b^Treatments compared adjusting for baseline post-bronchodilator percent predicted FEV_1_ and log baseline EOS count as continuous covariates and baseline COPD exacerbation history (0, 1, ≥ 2) in the preceding 12 months, region, and ICS use at screening (yes/no) as categorical covariates using negative binomial regression; time at risk of experiencing an exacerbation was used as an offset variable in the model

Abbreviations. BFF, budesonide/formoterol fumarate dihydrate; BGF, budesonide/glycopyrronium/formoterol fumarate dihydrate; COPD, chronic obstructive pulmonary disease; EOS, eosinophil; FEV_1_, forced expiratory volume in 1 s; GFF, glycopyrronium/formoterol fumarate dihydrate; ICS, inhaled corticosteroid; mITT, modified intention-to-treat; SE, standard error

Among patients with blood EOS count ≥ 100 cells/mm^3^, similar trends were observed in patients with blood EOS count 100 to < 300 cells/mm^3^ (Fig. 2C-D). However, this is not surprising as those with blood EOS count 100 to < 300 cells/mm^3^ constitute the majority of the sample; only 12.4% of patients in the KRONOS mITT had blood EOS count > 300 cells/mm^3^.

## Discussion

In this post-hoc analysis of the KRONOS study, lung function and exacerbation rates with BGF versus dual LAMA/LABA and ICS/LABA therapies were evaluated in patients with moderate-to-very severe COPD in blood EOS count subgroups, as a function of exacerbation history in the preceding 12 months and COPD severity. To the best of our knowledge, these are the first analyses to suggest that triple therapy is effective even in patients with no history of exacerbations and low levels of peripheral eosinophilia.

Triple therapy with BGF improved lung function, as measured by greater increases from baseline in morning pre-dose trough FEV_1_, versus dual ICS/LABA therapy with BFF, in patients with blood EOS count 100 to < 300 cells/mm^3^ without an exacerbation history in the preceding 12 months and among patients with moderate and severe COPD. Similar findings were observed among patients with blood EOS count ≥ 100 cells/mm^3^, which included a relatively small number of patients with blood EOS count ≥ 300 cells/mm^3^. Additionally, triple therapy with BGF reduced the annual moderate or severe exacerbations rate versus LAMA/LABA dual therapy with GFF in patients with blood EOS count 100 to < 300 cells/mm^3^ without an exacerbation history in the preceding 12 months and among those with moderate and severe COPD severity, with a similar trend observed for very severe COPD. Overall, these findings seem to indicate that benefits of triple BGF therapy versus dual LAMA/LABA and ICS/LABA therapy are observed across a range of blood EOS counts (even when blood EOS counts are 100 to < 300 cells/mm^3^) and exacerbation histories (including in the absence of exacerbations in the past year), and COPD severity (including those with moderate COPD). These findings may suggest that triple therapy with BGF is more effective than treatment without ICS, i.e., LAMA/LABA, in terms of exacerbations, and more effective than treatment without LAMA, i.e., ICS/LABA, in terms of lung function in some patients.

The observation that BGF conveys benefits over dual ICS/LABA and LAMA/LABA therapy in patients with blood EOS count 100 to < 300 cells/mm^3^ is consistent with previously published reports [9, 10, 20]. In post-hoc analyses of the 52-week ETHOS study, BGF improved morning pre-dose trough FEV_1_ versus BFF and GFF as well as reduced moderate or severe exacerbation rates versus GFF across a range of blood EOS counts (≥ 100, ≥ 100−<300, and ≥ 300 cells/mm³) [10]. In the KRONOS study, change from morning pre-dose trough FEV_1_ with BGF versus BFF and BUD/FORM, as well as reductions in the rate of moderate or severe exacerbations for BGF versus GFF, were observed in patients with blood EOS count < 150 cells/mm^3^ [9]. Similarly, results of the triple therapy studied in the 52-week IMPACT trial indicated that moderate or severe exacerbation rates with fluticasone furoate/umeclidinium/vilanterol triple therapy were lower compared with dual LAMA/LABA therapy with umeclidinium/vilanterol across a range of blood EOS levels, including at blood EOS count of approximately 100 to 300 cells/mm^3^ [20]. Although the duration of the intervention was not long enough, the reduction in exacerbation rate with BGF triple therapy may be considered clinically meaningful. The clinical significance of the improvement in respiratory function needs to be clarified in future studies.

In the KRONOS study, exacerbation history reported in the year before study entry was lower than the model-estimated rates observed during the study [9]. This suggests that there are other factors that lead to the risk of exacerbations, and not only exacerbation history in the preceding 12 months. Although, not having an exacerbation history in the preceding 12 months is not synonymous with reduced risk, it is widely accepted that those with a history of exacerbations are more likely to experience a future exacerbation [22]. This is supported by observations in the current analyses, as patients with an exacerbation history in the preceding 12 months before entering the study had numerically higher exacerbations rates during the study, irrespective of treatment arm or blood EOS level, compared with those without an exacerbation history in the preceding 12 months.

Current guidance in Japan recommends ICS/LAMA/LABA triple therapy in patients with COPD and no diagnosis of asthma who experience frequent exacerbations and have blood EOS count ≥ 300 cells/mm^3^, and in patients with COPD and features of asthma with continuing/worsening symptoms despite receiving dual ICS/LABA therapy [6]. Our analyses suggest BGF has beneficial effects on lung function versus dual ICS/LABA therapy and on moderate or severe exacerbation rates versus dual LAMA/LABA therapy in patients with and without recent exacerbation histories and among those with moderate and severe COPD who have blood EOS count 100 to < 300 cells/mm^3^. Similar results were generally observed for both exacerbation history and COPD severity in supportive analyses of patients with blood EOS count ≥ 100 cells/mm^3^ (i.e., when patients with blood EOS count > 300 cells/mm^3^ were included; BGF, *n* = 55; GFF, *n* = 56; BFF, *n* = 32). However, treatment differences on exacerbation rate reductions for BGF versus GFF did appear more robust in this subgroup in some instances, with beneficial effects observed in those with and without exacerbation histories. This is expected since a threshold of blood EOS count > 300 cells/mm^3^ identifies patients most likely to benefit from ICS [2].

ICS withdrawal has been raised as a concern in triple therapy studies among participants previously treated with an ICS who discontinued ICS following randomization to a non-ICS containing treatment arm [23]. In this regard, it is possible that those patients randomized to LAMA/LABA with GFF might have exhibited increased exacerbation rates due to removal of the ICS treatment component. However, a previously published post-hoc analysis of the ETHOS study, which examined the relationship between prior ICS use and benefits of BGF on exacerbations, symptoms, health-related quality of life, and lung function in patients with COPD, indicated there are benefits of BGF versus GFF regardless of ICS use within the 30 days before screening [24], suggesting ICS withdrawal may not account for the current findings.

Though the current findings seem to suggest benefits of ICS-containing triple therapy versus dual therapy on lung function and exacerbations, observations from a real-world observational study of triple therapy in COPD among ICS-naive patients highlight that triple therapy may have potential negative impacts, including increased incidence of severe pneumonia [25]. Other studies have also reported increased risk of other respiratory infections and pneumonia associated with ICS [26–28]. This emphasizes the importance of tailoring treatment plans to individual patient needs.

A few limitations of these analyses should be considered when interpreting these results. As the KRONOS study was not prospectively powered for any of the reported post-hoc analyses, reported *P*-values are nominal, unadjusted for multiplicity, and provided for descriptive purposes only. In addition, 74% of patients had no exacerbations in the last 12 months in the KRONOS study [9]. As such, sample sizes for post-hoc analyses of patients with an exacerbation history in the preceding 12 months were relatively small and subject to greater levels of variability. However, as the most compelling and clinically relevant findings from the perspective of current treatment guidelines relate to triple therapy use in patients without exacerbation history in the preceding 12 months, this limitation is not considered to be critical. It should be acknowledged that exacerbations are not a stable phenotype. Even though previous reports suggest the most important determinant and the singular predictive tool of frequent exacerbations is a history of exacerbations [29], there also patients who experience exacerbations in the previous year who do not experience exacerbations in the following year [29]. Therefore, when considering the exacerbation-suppressing effects of drug interventions, it is essential to consider the possibility some patients might not have experienced exacerbations even without drug intervention.

## Conclusions

In post-hoc analyses of patients with moderate-to-very severe COPD from the KRONOS study, benefits of ICS/LAMA/LABA triple therapy with BGF were observed for lung function versus dual ICS/LABA therapy, and for exacerbation rates versus dual LAMA/LABA therapy in patients with blood EOS count 100 to < 300 cells/mm^3^ who had less severe disease and no history of exacerbations in the last 12 months. Taken together, these data may suggest patients with blood EOS count > 100 cells/mm^3^ without a recent history of exacerbations and those with moderate disease could benefit from ICS/LAMA/LABA triple therapy with BGF relative to dual therapy with ICS/LABA or LAMA/LABA. Therefore, clinicians should consider a step-up to triple therapy in patients with persistent/worsening symptoms whose blood EOS count is ≥ 100 cells/mm^3^, even if overall disease severity is moderate and there is no recent history of exacerbations. However, these findings require confirmation in adequately controlled studies that are statistically powered to assess these endpoints.

### Electronic supplementary material

Below is the link to the electronic supplementary material.

Supplementary Material 1

## Data Availability

Data underlying the findings described in this manuscript may be obtained in accordance with AstraZeneca’s data sharing policy described at https://astrazenecagrouptrials.pharmacm.com/ST/Submission/Disclosure. Data for studies directly listed on Vivli can be requested through Vivli at www.vivli.org. Data for studies not listed on Vivli could be requested through Vivli at https://vivli.org/members/enquiries-about-studies-not-listed-on-the-vivli-platform/. The AstraZeneca Vivli member page is also available outlining further details: https://vivli.org/ourmember/astrazeneca/.

## Abbreviations

BFF: Budesonide/formoterol fumarate dihydrate
BGF: Budesonide/glycopyrronium/formoterol fumarate dihydrate
BUD/FORM: Budesonide/formoterol fumarate dihydrate (via dry-powder inhaler)
CI: Confidence interval
COPD: Chronic obstructive pulmonary disease
EOS: Eosinophil
FEV_1_: Forced expiratory volume in 1 s
GOLD: Global initiative for Chronic Obstructive Lung disease
GFF: Glycopyrronium/formoterol fumarate dihydrate
ICS: Inhaled corticosteroid
LABA: Long-acting β_2_-agonist
LAMA: Long-acting muscarinic antagonist
LS: Least squares
mITT: Modified intention-to-treat
RR: Rate ratio
SE: Standard error
