# Supplementary material for: Benefits of budesonide/glycopyrronium/formoterol fumarate dihydrate on lung function and exacerbations of COPD: a post-hoc analysis of the KRONOS study by blood eosinophil level and exacerbation history
Source: Respir Res. 2024 Aug 5;25:297. doi: 10.1186/s12931-024-02918-8 (PMC11302094; doi:10.1186/s12931-024-02918-8)
Supplement: Supplementary file 1 — Supplementary Material 1 [file 12931_2024_2918_MOESM1_ESM.docx]

# Additional files

## Additional file 1 Table S1 Demographic and clinical characteristics: EOS count 100 to <300 cells/mm^3^ by COPD severity, mITT population

|  | **BGF**  **320/14.4/10 μg** | **GFF**  **14.4/10 μg** | **BFF**  **320/10 μg** |
| --- | --- | --- | --- |
| **EOS 100 to <300 cells/mm^3^ – Moderate COPD** | **N = 201** | **N = 204** | **N = 90** |
| **Mean (SD) age, years^a^** | 64.4 (8.8) | 64.4 (8.2) | 65.4 (7.4) |
| **Sex, n (%) male** | 138 (68.7) | 127 (62.3) | 59 (65.6) |
| **EOS count** |  |  |  |
| Median (range) cells per mm^3^ | 175.0  (100.0–295.0) | 165.0  (100.0–295.0) | 175.0  (100.0–273.3) |
| ≥150 cells/mm^3^, n (%) | 128 (63.7) | 135 (66.2) | 62 (68.9) |
| **Current smoker, n (%)** | 91 (45.3) | 88 (43.1) | 41 (45.6) |
| **Mean (SD) number of pack-years smoked^b^** | 52.6 (30.7) | 50.2 (28.2) | 50.0 (25.4) |
| **Mean (SD) post-salbutamol FEV_1_, % predicted** | 62.2 (8.1) | 61.9 (8.0) | 62.7 (7.7) |
| **Moderate or severe COPD exacerbations in the past 12 months, n (%)** |  |  |  |
| 0 | 157 (78.1) | 166 (81.4) | 70 (77.8) |
| 1 | 29 (14.4) | 28 (13.7) | 16 (17.8) |
| ≥2 | 15 (7.5) | 10 (4.9) | 4 (4.4) |
| **Used ICS at screening, n (%)** | 132 (65.7) | 135 (66.2) | 63 (70.0) |
| **COPD severity, n (%)** |  |  |  |
| Mild | 0 | 0 | 0 |
| Moderate | 201 (100.0) | 204 (100.0) | 90 (100.0) |
| Severe | 0 | 0 | 0 |
| Very severe | 0 | 0 | 0 |
| **Mean (SD) total CAT score^c^** | 18.5 (6.6) | 17.7 (6.4) | 18.1 (6.6) |
| **Reversibility^d^, n (%)** | 98 (48.8) | 100 (49.0) | 47 (52.2) |
| **EOS 100 to <300 cells/mm^3^ – Severe COPD** | **N = 182** | **N = 163** | **N = 89** |
| **Mean (SD) age, years^a^** | 64.5 (7.8) | 65.6 (7.2) | 64.9 (7.3) |
| **Sex, n (%) male** | 118 (64.8) | 114 (69.9) | 68 (76.4) |
| **EOS count** |  |  |  |
| Median (range) cells per mm^3^ | 160.0  (100.0–295.0) | 155.0  (100.0–295.0) | 175.0  (100.0–295.0) |
| ≥150 cells/mm^3^, n (%) | 111 (61.0) | 94 (57.7) | 55 (61.8) |
| **Current smoker, n (%)** | 74 (40.7) | 68 (41.7) | 33 (37.1) |
| **Mean (SD) number of pack-years smoked^b^** | 55.5 (31.8) | 51.4 (24.4) | 54.3 (32.0) |
| **Mean (SD) post-salbutamol FEV_1_, % predicted** | 41.1 (5.5) | 41.2 (6.1) | 40.1 (5.5) |
| **Moderate or severe COPD exacerbations in the past 12 months, n (%)** |  |  |  |
| 0 | 131 (72.0) | 121 (74.2) | 70 (78.7) |
| 1 | 42 (23.1) | 31 (19.0) | 14 (15.7) |
| ≥2 | 9 (4.9) | 11 (6.7) | 5 (5.6) |
| **Used ICS at screening, n (%)** | 135 (74.2) | 112 (68.7) | 64 (71.9) |
| **COPD severity, n (%)** |  |  |  |
| Mild | 0 | 0 | 0 |
| Moderate | 0 | 0 | 0 |
| Severe | 182 (100.0) | 163 (100.0) | 89 (100.0) |
| Very severe | 0 | 0 | 0 |
| **Mean (SD) total CAT score (0–40)^c^** | 18.7 (6.0) | 18.7 (6.0) | 19.4 (6.4) |
| **Reversibility^d^, n (%)** | 83 (45.6) | 60 (36.8) | 35 (39.3) |
| **EOS 100 to <300 cells/mm^3^ – Very severe COPD** | **N = 26** | **N = 32** | **N = 12** |
| **Mean (SD) age, years^a^** | 66.5 (4.8) | 64.0 (7.8) | 65.8 (6.2) |
| **Sex, n (%) male** | 17 (65.4) | 19 (59.4) | 8 (66.7) |
| **EOS count** |  |  |  |
| Median (range) cells per mm^3^ | 155.0  (100.0–275.0) | 178.3  (105.0–290.0) | 145.0  (100.0–270.0) |
| ≥150 cells/mm^3^, n (%) | 14 (53.8) | 20 (62.5) | 6 (50.0) |
| **Current smoker, n (%)** | 12 (46.2) | 12 (37.5) | 3 (25.0) |
| **Mean (SD) number of pack-years smoked^b^** | 63.9 (27.9) | 51.9 (28.9) | 53.5 (25.4) |
| **Mean (SD) post-salbutamol FEV_1_, % predicted** | 27.4 (1.8) | 27.4 (1.5) | 28.0 (1.3) |
| **Moderate or severe COPD exacerbations in the past 12 months, n (%)** |  |  |  |
| 0 | 20 (76.9) | 20 (62.5) | 6 (50.0) |
| 1 | 5 (19.2) | 8 (25.0) | 4 (33.3) |
| ≥2 | 1 (3.8) | 4 (12.5) | 2 (16.7) |
| **Used ICS at screening, n (%)** | 16 (61.5) | 26 (81.3) | 10 (83.3) |
| **COPD severity, n (%)** |  |  |  |
| Mild | 0 | 0 | 0 |
| Moderate | 0 | 0 | 0 |
| Severe | 0 | 0 | 0 |
| Very severe | 26 (100.0) | 32 (100.0) | 12 (100.0) |
| **Mean (SD) total CAT score^c^** | 22.8 (7.7) | 20.6 (6.1) | 20.1 (8.2) |
| **Reversibility^d^, n (%)** | 6 (23.1) | 2 (6.3) | 4 (33.3) |

^a^Age is the age at the time of informed consent.

^b^(Number of cigarettes per day / 20) × number of years smoked.

^c^The total score is the sum of eight CAT item scores.

^d^Reversibility defined as improvement in FEV_1_ after salbutamol administration (compared with before salbutamol administration) of 12% or more and 200 mL or more.

Abbreviations. BFF: budesonide/formoterol fumarate dihydrate; BGF: budesonide/glycopyrronium/formoterol fumarate dihydrate; CAT: COPD Assessment Test; COPD: chronic obstructive pulmonary disease; EOS: eosinophil; FEV_1_: forced expiratory volume in 1 second; GFF: glycopyrronium/formoterol fumarate dihydrate; ICS, inhaled corticosteroid; mITT: modified intention-to-treat; SD: standard deviation.

## Additional file 1 Table S2 Demographic and clinical characteristics: EOS count ≥100 cells/mm^3^ by exacerbation history, mITT population

|  | **BGF**  **320/14.4/10 μg** | **GFF**  **14.4/10 μg** | **BFF**  **320/10 μg** |
| --- | --- | --- | --- |
| **EOS ≥100** **cells/mm^3^ – without exacerbation history in the preceding 12 months** | **N = 363** | **N = 368** | **N = 179** |
| **Mean (SD) age, years^a^** | 65.5 (7.8) | 65.2 (7.8) | 65.9 (7.1) |
| **Sex, n (%) male** | 249 (68.6) | 258 (70.1) | 132 (73.7) |
| **EOS count** |  |  |  |
| Median (range) cells per mm^3^ | 180.0  (100.0–2815.0) | 182.5  (100.0–2490.0) | 200.0  (100.0–920.0) |
| ≥150 cells/mm^3^, n (%) | 245 (67.5) | 249 (67.7) | 132 (73.7) |
| **Current smoker, n (%)** | 157 (43.3) | 153 (41.6) | 67 (37.4) |
| **Mean (SD) number of pack-years smoked^b^** | 55.9 (33.1) | 51.7 (27.2) | 51.9 (25.8) |
| **Mean (SD) post-salbutamol FEV_1_, % predicted** | 51.6 (13.8) | 51.8 (13.4) | 50.5 (13.4) |
| **Moderate or severe COPD exacerbations in the past 12 months, n (%)** |  |  |  |
| 0 | 363 (100) | 368 (100) | 179 (100) |
| 1 | 0 | 0 | 0 |
| ≥2 | 0 | 0 | 0 |
| **Used ICS at screening, n (%)** | 251 (69.1) | 253 (68.8) | 125 (69.8) |
| **COPD severity, n (%)** |  |  |  |
| Mild | 1 (0.3) | 0 | 1 (0.6) |
| Moderate | 190 (52.3) | 199 (54.1) | 86 (48.0) |
| Severe | 149 (41.0) | 147 (39.9) | 84 (46.9) |
| Very severe | 23 (6.3) | 22 (6.0) | 8 (4.5) |
| **Mean (SD) total CAT score ^c^** | 18.8 (6.7) | 18.2 (6.1) | 18.7 (6.9) |
| **Reversibility^d^, n (%)** | 165 (45.5) | 167 (45.4) | 79 (44.1) |
| **EOS ≥100** **cells/mm^3^ – with exacerbation history in the preceding 12 months** | **N = 119** | **N = 116** | **N = 53** |
| **Mean (SD) age, years^a^** | 63.1 (8.9) | 64.8 (8.1) | 62.9 (7.8) |
| **Sex, n (%) male** | 81 (68.1) | 71 (61.2) | 31 (58.5) |
| **EOS count** |  |  |  |
| Median (range) cells per mm^3^ | 180.0  (100.0–1290.0) | 190.0  (105.0–790.0) | 165.0  (100.0–725.0) |
| ≥150 cells/mm^3^, n (%) | 80 (67.2) | 85 (73.3) | 31 (58.5) |
| **Current smoker, n (%)** | 50 (42.0) | 46 (39.7) | 23 (43.4) |
| **Mean (SD) number of pack-years smoked^b^** | 47.9 (26.4) | 49.2 (24.9) | 54.6 (35.2) |
| **Mean (SD) post-salbutamol FEV_1_, % predicted** | 50.2 (13.4) | 48.5 (14.4) | 50.8 (15.8) |
| **Moderate or severe COPD exacerbations in the past 12 months, n (%)** |  |  |  |
| 0 | 0 | 0 | 0 |
| 1 | 90 (75.6) | 83 (71.6) | 40 (75.5) |
| ≥2 | 29 (24.4) | 33 (28.4) | 13 (24.5) |
| **Used ICS at screening, n (%)** | 89 (74.8) | 86 (74.1) | 42 (79.2) |
| **COPD severity, n (%)** |  |  |  |
| Mild | 1 (0.8) | 0 | 0 |
| Moderate | 55 (46.2) | 51 (44.0) | 27 (50.9) |
| Severe | 56 (47.1) | 51 (44.0) | 20 (37.7) |
| Very severe | 7 (5.9) | 14 (12.1) | 6 (11.3) |
| **Mean (SD) total CAT score ^c^** | 19.7 (6.5) | 18.8 (6.5) | 19.1 (6.6) |
| **Reversibility^d^, n (%)** | 60 (50.4) | 38 (32.8) | 24 (45.3) |

^a^Age is the age at the time of informed consent.

^b^(Number of cigarettes per day / 20) × number of years smoked.

^c^The total score is the sum of eight CAT item scores.

^d^Reversibility defined as improvement in FEV_1_ after salbutamol administration (compared with before salbutamol administration) of 12% or more and 200 mL or more.

Abbreviations. BFF: budesonide/formoterol fumarate dihydrate; BGF: budesonide/glycopyrronium/formoterol fumarate dihydrate; CAT: COPD Assessment Test; COPD: chronic obstructive pulmonary disease; EOS: eosinophil; FEV_1_: forced expiratory volume in 1 second; GFF: glycopyrronium/formoterol fumarate dihydrate; ICS, inhaled corticosteroid; mITT: modified intention-to-treat; SD: standard deviation.

## Additional file 1 Table S3 Demographic and clinical characteristics: EOS count ≥100 cells/mm^3^ by COPD severity, mITT population

|  | **BGF**  **320/14.4/10 μg** | **GFF**  **14.4/10 μg** | **BFF**  **320/10 μg** |
| --- | --- | --- | --- |
| **EOS ≥100 cells/mm^3^ – Moderate COPD** | **N = 245** | **N = 250** | **N = 113** |
| **Mean (SD) age, years^a^** | 65.0 (8.6) | 64.6 (8.3) | 65.2 (7.5) |
| **Sex, n (%) male** | 173 (70.6) | 166 (66.4) | 74 (65.5) |
| **EOS count** |  |  |  |
| Median (range) cells per mm^3^ | 190.0  (100.0–2815.0) | 185.0  (100.0–2490.0) | 190.0  (100.0–920.0) |
| ≥150 cells/mm^3^, n (%) | 172 (70.2) | 181 (72.4) | 85 (75.2) |
| **Current smoker, n (%)** | 103 (42.0) | 101 (40.4) | 48 (42.5) |
| **Mean (SD) number of pack-years smoked^b^** | 52.1 (32.2) | 50.5 (27.8) | 50.0 (25.1) |
| **Mean (SD) post-salbutamol FEV_1_, % predicted** | 62.1 (8.0) | 62.0 (8.0) | 62.4 (7.7) |
| **Moderate or severe COPD exacerbations in the past 12 months, n (%)** |  |  |  |
| 0 | 190 (77.6) | 199 (79.6) | 86 (76.1) |
| 1 | 37 (15.1) | 36 (14.4) | 21 (18.6) |
| ≥2 | 18 (7.3) | 15 (6.0) | 6 (5.3) |
| **Used ICS at screening, n (%)** | 165 (67.3) | 169 (67.6) | 79 (69.9) |
| **COPD severity, n (%)** |  |  |  |
| Mild | 0 | 0 | 0 |
| Moderate | 245 (100.0) | 250 (100.0) | 113 (100.0) |
| Severe | 0 | 0 | 0 |
| Very severe | 0 | 0 | 0 |
| **Mean (SD) total CAT score^c^** | 18.5 (6.6) | 17.5 (6.3) | 18.0 (6.7) |
| **Reversibility^d^, n (%)** | 124 (50.6) | 127 (50.8) | 59 (52.2) |
| **EOS ≥100 cells/mm^3^ – Severe COPD** | **N = 205** | **N = 198** | **N = 104** |
| **Mean (SD) age, years^a^** | 64.4 (7.9) | 65.8 (7.4) | 65.1 (7.3) |
| **Sex, n (%) male** | 134 (65.4) | 140 (70.7) | 79 (76.0) |
| **EOS count** |  |  |  |
| Median (range) cells per mm^3^ | 170.0  (100.0–670.0) | 187.5  (100.0–2465.0) | 200.0  (100.0–520.0) |
| ≥150 cells/mm^3^, n (%) | 134 (65.4) | 129 (65.2) | 70 (67.3) |
| **Current smoker, n (%)** | 88 (42.9) | 86 (43.4) | 39 (37.5) |
| **Mean (SD) number of pack-years smoked^b^** | 54.7 (31.1) | 51.7 (24.9) | 54.4 (31.1) |
| **Mean (SD) post-salbutamol FEV_1_, % predicted** | 41.3 (5.5) | 41.5 (5.9) | 40.4 (5.8) |
| **Moderate or severe COPD exacerbations in the past 12 months, n (%)** |  |  |  |
| 0 | 149 (72.7) | 147 (74.2) | 84 (80.8) |
| 1 | 46 (22.4) | 39 (19.2) | 15 (14.4) |
| ≥2 | 10 (4.9) | 13 (6.6) | 5 (4.8) |
| **Used ICS at screening, n (%)** | 154 (75.1) | 141 (71.2) | 75 (72.1) |
| **COPD severity, n (%)** |  |  |  |
| Mild | 0 | 0 | 0 |
| Moderate | 0 | 0 | 0 |
| Severe | 205 (100.0) | 198 (100.0) | 104 (100.0) |
| Very severe | 0 | 0 | 0 |
| **Mean (SD) total CAT score (0–40)^c^** | 19.2 (6.4) | 19.1 (6.0) | 19.5 (6.7) |
| **Reversibility^d^, n (%)** | 93 (45.4) | 76 (38.4) | 40 (38.5) |
| **EOS ≥100 cells/mm^3^ – Very severe COPD** | **N = 30** | **N = 36** | **N = 14** |
| **Mean (SD) age, years^a^** | 66.7 (5.4) | 64.6 (7.6) | 64.8 (6.2) |
| **Sex, n (%) male** | 21 (70.0) | 23 (63.9) | 9 (64.3) |
| **EOS count** |  |  |  |
| Median (range) cells per mm^3^ | 170.0  (100.0–565.0) | 187.5  (105.0–390.0) | 155.0  (100.0–395.0) |
| ≥150 cells/mm^3^, n (%) | 18 (60.0) | 24 (66.7) | 8 (57.1) |
| **Current smoker, n (%)** | 15 (50.0) | 12 (33.3) | 3 (21.4) |
| **Mean (SD) number of pack-years smoked^b^** | 65.3 (29.8) | 52.3 (28.3) | 54.8 (24.8) |
| **Mean (SD) post-salbutamol FEV_1_, % predicted** | 27.6 (1.7) | 27.5 (1.5) | 28.1 (1.3) |
| **Moderate or severe COPD exacerbations in the past 12 months, n (%)** |  |  |  |
| 0 | 23 (76.7) | 22 (61.1) | 8 (57.1) |
| 1 | 6 (20.0) | 9 (25.0) | 4 (28.6) |
| ≥2 | 1 (3.3) | 5 (13.9) | 2 (14.3) |
| **Used ICS at screening, n (%)** | 20 (66.7) | 29 (80.6) | 12 (85.7) |
| **COPD severity, n (%)** |  |  |  |
| Mild | 0 | 0 | 0 |
| Moderate | 0 | 0 | 0 |
| Severe | 0 | 0 | 0 |
| Very severe | 30 (100.0) | 36 (100.0) | 14 (100.0) |
| **Mean (SD) total CAT score^c^** | 22.9 (7.4) | 20.5 (5.9) | 19.9 (7.8) |
| **Reversibility^d^, n (%)** | 7 (23.3) | 2 (5.6) | 4 (28.6) |

^a^Age is the age at the time of informed consent.

^b^(Number of cigarettes per day / 20) × number of years smoked.

^c^The total score is the sum of eight CAT item scores.

^d^Reversibility defined as improvement in FEV_1_ after salbutamol administration (compared with before salbutamol administration) of 12% or more and 200 mL or more.

Abbreviations. BFF: budesonide/formoterol fumarate dihydrate; BGF: budesonide/glycopyrronium/formoterol fumarate dihydrate; CAT: COPD Assessment Test; COPD: chronic obstructive pulmonary disease; EOS: eosinophil; FEV_1_: forced expiratory volume in 1 second; GFF: glycopyrronium/formoterol fumarate dihydrate; ICS: inhaled corticosteroid: mITT: modified intention-to-treat; SD: standard deviation.

## Additional file 1 Table S4 Lung function^a^: EOS subgroups by exacerbation history and COPD severity, mITT population

|  | **BGF**  **320/14.4/10 μg** | **GFF**  **14.4/10 μg** | **BFF**  **320/10 μg** |
| --- | --- | --- | --- |
| **EOS 100 to <300 cells/mm^3^** |  |  |  |
| **Without exacerbation history in the preceding 12 months** | **N = 308** | **N = 307** | **N = 147** |
| Mean ± SD baseline FEV_1_ (L)^b^ | 1.217±0.467 (n=284) | 1.237±0.436 (n=272) | 1.191±0.399 (n=131) |
| LS mean (SE) change from baseline^c^ | 0.127 (0.0101) | 0.131 (0.0102) | 0.053 (0.0147) |
| **With exacerbation history in the preceding 12 months** | **N = 102** | **N = 92** | **N = 45** |
| Mean ± SD baseline FEV_1_ (L)^b^ | 1.157±0.474 (n=97) | 1.056±0.410 (n=82) | 1.141±0.501 (n=38) |
| LS mean (SE) change from baseline^c^ | 0.125 (0.0159) | 0.128 (0.0169) | 0.082 (0.0244) |
| **Moderate COPD** | **N = 201** | **N = 204** | **N = 90** |
| Mean ± SD baseline FEV_1_ (L)^b^ | 1.509±0.424 (n=188) | 1.442±0.379 (n=188) | 1.464±0.393 (n=78) |
| LS mean (SE) change from baseline^c^ | 0.127 (0.0128) | 0.130 (0.0127) | 0.057 (0.0194) |
| **Severe COPD** | **N = 182** | **N = 163** | **N = 89** |
| Mean ± SD baseline FEV_1_ (L)^b^ | 0.934±0.261 (n=170) | 0.964±0.307 (n=139) | 0.971±0.243 (n=79) |
| LS mean (SE) change from baseline^c^ | 0.125 (0.0122) | 0.132 (0.0131) | 0.063 (0.0175) |
| **Very severe COPD** | **N = 26** | **N = 32** | **N = 12** |
| Mean ± SD baseline FEV_1_ (L)^b^ | 0.612±0.156 (n=22) | 0.665±0.181 (n=27) | 0.618±0.142 (n=11) |
| LS mean (SE) change from baseline^c^ | 0.148 (0.0305) | 0.117 (0.0282) | 0.066 (0.0449) |
| **EOS ≥100** **cells/mm^3^** |  |  |  |
| **Without exacerbation history in the preceding 12 months** | **N = 363** | **N = 368** | **N = 179** |
| Mean ± SD baseline FEV_1_ (L)^b^ | 1.222±0.454 (n=336) | 1.251±0.443 (n=325) | 1.209±0.403 (n=159) |
| LS mean (SE) change from baseline^c^ | 0.130 (0.0097) | 0.120 (0.0097) | 0.049 (0.0138) |
| **With exacerbation history in the preceding 12 months** | **N = 119** | **N = 116** | **N = 53** |
| Mean ± SD baseline FEV_1_ (L)^b^ | 1.194±0.469 (n=113) | 1.084±0.409 (n=103) | 1.160±0.469 (n=46) |
| LS mean (SE) change from baseline^c^ | 0.145 (0.0167) | 0.108 (0.0172) | 0.131 (0.0251) |
| **Moderate COPD** | **N = 245** | **N = 250** | **N = 113** |
| Mean ± SD baseline FEV_1_ (L)^b^ | 1.497±0.408 (n=230) | 1.463±0.380 (n=228) | 1.459±0.379 (n=99) |
| LS mean (SE) change from baseline^c^ | 0.139 (0.0125) | 0.110 (0.0124) | 0.074 (0.0186) |
| **Severe COPD** | **N = 205** | **N = 198** | **N = 104** |
| Mean ± SD baseline FEV_1_ (L)^b^ | 0.946±0.262 (n=192) | 0.969±0.309 (n=169) | 0.993±0.264 (n=92) |
| LS mean (SE) change from baseline^c^ | 0.126 (0.0119) | 0.122 (0.0124) | 0.064 (0.0167) |
| **Very severe COPD** | **N = 30** | **N = 36** | **N = 14** |
| Mean ± SD baseline FEV_1_ (L)^b^ | 0.627±0.162 (n=25) | 0.681±0.179 (n=31) | 0.630±0.135 (n=13) |
| LS mean (SE) change from baseline^c^ | 0.143 (0.0299) | 0.106 (0.0275) | 0.031 (0.0432) |

^a^Change from baseline in morning pre-dose trough FEV_1_ over 12–24 weeks.

^b^Baseline defined as the mean of all evaluable 60- and 30-minute pre-dose values on Day 1 (Visit 4).

^c^LS mean is from a linear repeated measures model which included the following covariates: baseline FEV_1_, percent reversibility to salbutamol, and baseline EOS count as continuous covariates and visit, treatment, the treatment-by-visit interaction, and ICS use at screening (yes/no) as categorical covariates.

Abbreviations. BFF: budesonide/formoterol fumarate dihydrate; BGF: budesonide/glycopyrronium/formoterol fumarate dihydrate; COPD: chronic obstructive pulmonary disease; EOS: eosinophil; FEV_1_: forced expiratory volume in 1 second; GFF: glycopyrronium/formoterol fumarate dihydrate; ICS: inhaled corticosteroid; LS: least squares; mITT: modified intention-to-treat; SD: standard deviation; SE: standard error.
